# rhBMP-2-loaded hydroxyapatite/beta-tricalcium phosphate microsphere/hydrogel composite promotes bone regeneration in a novel rat femoral nonunion model

**DOI:** 10.3389/fbioe.2024.1461260

**Published:** 2024-10-07

**Authors:** Takayuki Kitahara, Daisuke Tateiwa, Hiromasa Hirai, Masato Ikuta, Takuya Furuichi, Masayuki Bun, Yuichiro Ukon, Yuya Kanie, Masayuki Furuya, Takahito Fujimori, Seiji Okada, Takashi Kaito

**Affiliations:** ^1^ Department of Orthopaedic Surgery, Osaka University Graduate School of Medicine, Osaka, Japan; ^2^ Department of Orthopaedic Surgery, Osaka General Medical Center, Osaka, Japan; ^3^ Department of Orthopaedic Surgery, Osaka Rosai Hospital, Osaka, Japan

**Keywords:** nonunion, bone morphogenetic protein, bone regeneration, biomaterial, HA/β-TCP/hydrogel

## Abstract

**Background:**

Nonunion following fracture treatment remains a significant clinical challenge, adversely affecting the patient’s quality of life and imposing a substantial economic burden. The emergence of bone morphogenetic protein 2 (BMP-2) for bone regeneration represents a promising avenue, albeit limited by side effects such as inflammatory reactions primarily due to suboptimal drug delivery systems. This study focuses on NOVOSIS putty (NP), a novel biomaterial designed for the sustained release of BMP-2, aiming to mitigate these limitations and enhance bone healing.

**Objective:**

This research aimed to evaluate the effectiveness of NP, a hydroxyapatite granules/β-tricalcium phosphate hydrogel composite (HA/β-TCP/hydrogel), as a BMP-2 carrier for promoting bone regeneration in a new rat nonunion model of long bone.

**Methods:**

Using Sprague Dawley rats, a 2-mm silicone disk was interposed at the femoral fracture site, and intramedullary fixation with K-wire was performed to create a nonunion with a 2-mm bone defect. After 3 weeks, internal fixation with a plate, removal of the silicon disk, and refreshing the nonunion site were performed by implanting three different materials into the nonunion sites: allogenic iliac bone (IB), collagen sponge (CS) containing 10 μg of BMP-2, or NP containing 10 μg of BMP-2. Bone healing was evaluated weekly using micro-computed tomography (CT); *ex vivo* micro-Ct and histological evaluation were conducted at 6 weeks.

**Results:**

At 6 weeks, NP demonstrated a significantly higher bone union rate (76.5%) compared with the CS group (35.3%, *p* = 0.037), and the IB group (6.3%, *p* < 0.0001). Bone mineral density (BMD) and bone volume/tissue volume (BV/TV) were also significantly higher in the NP group compared with the CS group (BMD, *p* < 0.0001; BV/TV, *p* = 0.031). Histological analysis showed the fracture gap in the NP group was filled with more trabecular bone and less fibrous tissue compared with the CS group.

**Conclusion:**

The study confirms NP is a highly effective BMP-2 carrier, significantly improving bone union rates and new bone formation in nonunion fractures. The sustained release of BMP-2 from the hydrogel component reduced inflammatory responses and enhanced bone regeneration. NP can be a promising alternative to collagen-based BMP-2 delivery systems.

## 1 Introduction

Nonunion, which occurs in 5%–10% of fracture treatments (Canadian Orthopaedic Trauma Society, 2003; Mills and Simpson, 2013) is a significant problem that lowers the quality of life and increases the financial burden on patients (Borrelli et al., 2003; Bozic et al., 2003; Zura et al., 2016; Wildemann et al., 2021). For example, nonunion of long bones such as the femur or tibia can result in prolonged disability, chronic pain, and repeated surgical interventions, leading to substantial healthcare costs and loss of productivity (Nicholson et al., 2020; Rupp et al., 2018). The standard treatment is autologous bone grafting but, even with advanced techniques, approximately 17%–44% of cases do not achieve bone fusion (Zimmermann et al., 2009; Flierl et al., 2013; Azi et al., 2016). Additionally, autologous bone grafting has limitations due to pain at the donor site and limited availability of bone (Younger and Chapman, 1989; Banwart et al., 1995; Arrington et al., 1996; Ebraheim et al., 2001).

One potential treatment is bone regeneration therapy using bone morphogenetic protein 2 (BMP-2), a strong inducer of bone formation (Urist, 1965; Wozney et al., 1988; Spector et al., 2001). However, current BMP-2 products, which use collagen sponges (CS), have side effects such as local inflammation and ectopic bone formation, probably due to the rapid release of BMPs (Friess et al., 1999; Schmidmaier et al., 2007; Kowalczewski and Saul, 2018).

To solve these problems, we developed NOVOSIS putty (NP), a new biomaterial that slowly releases BMP-2 to reduce side effects and uses hydroxyapatite (HA) and beta-tricalcium phosphate (β-TCP) as a scaffold for efficient bone regeneration (Tateiwa et al., 2021; Nakagawa et al., 2022). NP is made of HA granules, β-TCP microspheres/poloxamer 407-based hydrogel (Veyries et al., 1999; Dumortier et al., 2006; Giuliano et al., 2020; β-TCP/hydrogel), and recombinant human BMP-2 (rhBMP-2). We previously reported the effects of NP on enhancing spinal fusion rates and reducing side effects using a rat model of posterolateral spinal fusion and interbody spinal fusion (Tateiwa et al., 2021; Nakagawa et al., 2022).

To evaluate the effects of NP in nonunion, we developed a new rat nonunion model (Tateiwa et al., 2024). Our newly developed rat nonunion model was specifically designed to mimic the clinical scenario of long bone nonunion more accurately than previous models. Traditional animal nonunion models, such as the periosteal cauterization model (Kokubu et al., 2003) and large bone defect model (Sato et al., 2014), primarily involve a single-stage surgical procedure. These models often mimic acute fractures and fail to replicate the complex biological environment and mechanical instability associated with chronic nonunion conditions. This limitation results in a focus on the early phases of fracture healing without addressing the chronic nature of nonunion. Our new model addresses these limitations by incorporating a two-stage surgical procedure and a silicone disk to create a reproducible and clinically relevant nonunion environment.

The purpose of this study was to compare the effects of NP with BMP-2, CS with BMP-2, and iliac bone (IB) grafting in a rat femoral nonunion model.

## 2 Materials and methods

### 2.1 Characteristics of HA and β-TCP components in the HA/β-TCP/hydrogel composite

**FIGURE 1:**
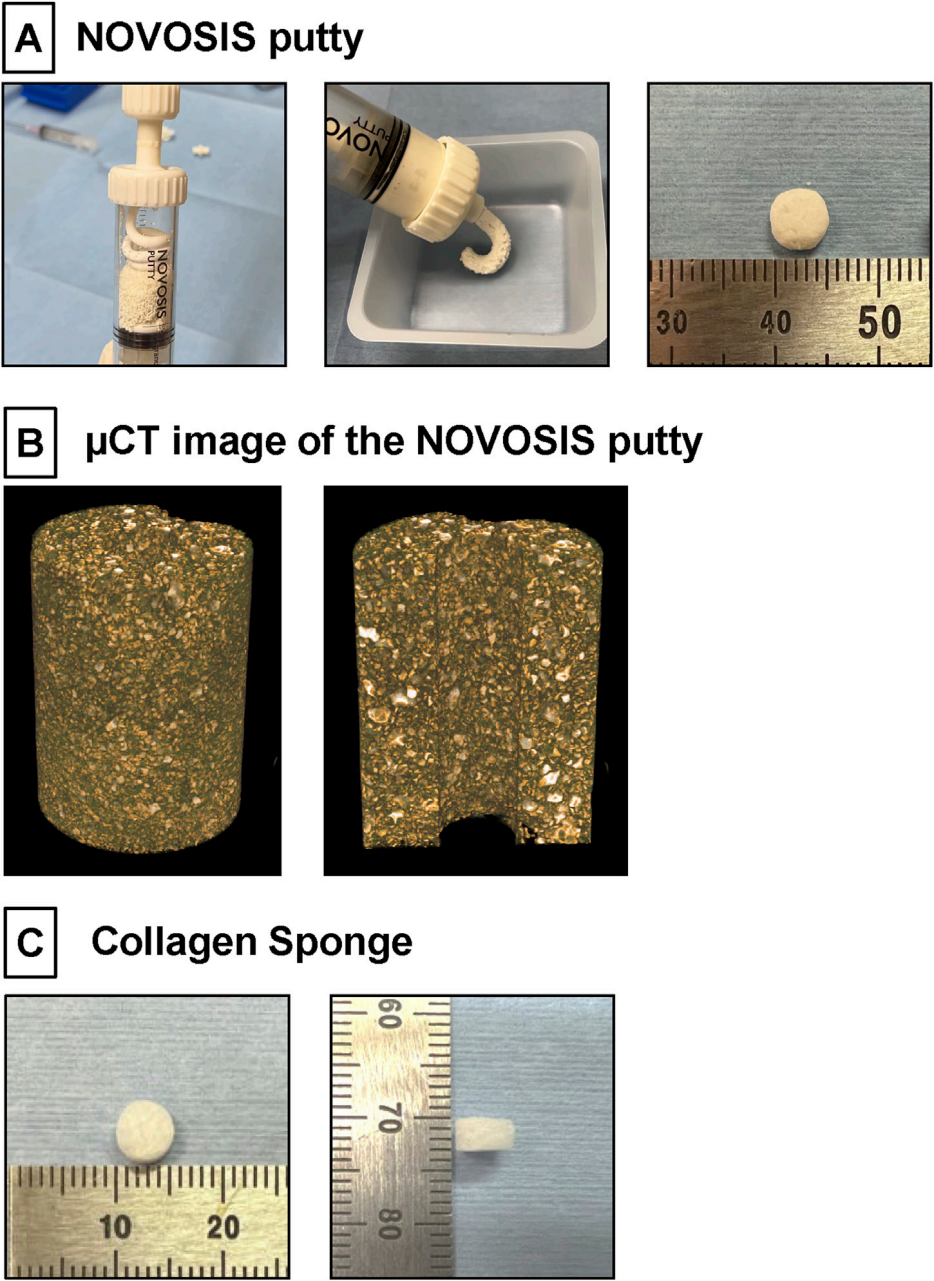
**(A)** NOVOSIS putty (NP). Hydroxyapatite granules and β-TCP microsphere/poloxamer 407-based hydrogel are mixed in a syringe. Composite is injectable and moldable. These composites were molded to a volume of 100 mm³ (80 mg). **(B)** µCT image showing the distribution of ceramic and hydrogel in the NP blended formulation. The final composition of NP consists of two sizes of ceramic particles, which appear white, were observed within the hydrogel. **(C)** Collagen Sponge. A disk-shaped collagen sponge (8.0 mm in diameter and 3 mm thick) was prepared by punching out an absorbable collagen sponge (CS; Colla Tape, Zimmer Dental, Carlsbad, CA, United States).

NP, a HA/β-TCP/hydrogel composite comprising 40% HA granulates and 60% β-TCP, was kindly provided by CGBio Co., Ltd. (Seoul, South Korea) (Figure 1A). The HA granules were 3.0–6.0 mm in size, with a porosity of approximately 70% and pore interconnectivity of 99%. Spray-dried β-TCP microspheres were approximately 45–75 μm in size and exhibited approximately 68% porosity. The hydrogel, biodegradable and biocompatible poloxamer 407, was combined with β-TCP microspheres at a 1:1 weight ratio. In addition to assessing their general appearance, the detailed microstructures of β-TCP/hydrogel and HA were determined using µCT image (Figure 1B). The X-ray diffractometer (XRD) patterns of HA and β-TCP matched the theoretical XRD patterns specified by the International Centre for Diffraction Data (ICDD) (Supplementary Figure 1).

The *in vitro* and *in vivo* rhBMP-2 release profile of NP has been described in detail in a previous report (Tateiwa et al., 2021). In that study, the *in vitro* release kinetics showed that until day 24, CS released 78.4% of the initially loaded dose (4 μg), while NP released 22.5% of the initial dose. From days 1–7, CS released the majority of its rhBMP-2, with the release tapering off thereafter. In contrast, NP exhibited a more controlled release pattern, with higher amounts being released between days 7–14 and days 14–24 compared to CS. *In vivo*, the biological half-life of rhBMP-2 was 6.2 h in NP compared to 3.8 h in CS.

### 2.2 Animal study groups

#### 2.2.1 Ethical approval and grouping

All animal experiments were approved by the Animal Experimental Committee of our institution (approval number: 04-078-001) and were conducted in strict accordance with the NIH Guide for the Care and Use of Laboratory Animals (Committee for the Update of the Guide for theUse of Laboratory Animals, 2011), and the study’s reporting adheres to the ARRIVE guidelines. Fifty-four male Sprague Dawley rats, 8 weeks old (Charles River Laboratories, Japan Inc., Kanagawa, Japan), weighing 220–250 g, were used for this study. These rats were divided into three groups based on the grafting materials used: allogenic iliac bone (IB group), collagen sponge (CS) soaked with 10 μg of rhBMP-2 (CS group), and HA/β-TCP hydrogel composite with 10 μg of rhBMP-2 (NP group) (Table 1). When an unintentional femoral fracture occurred during the plate fixation procedure, a screw was completely dislocated from the bone during *in vivo* CT follow-up, or a rat developed a wound infection, the rat was immediately euthanized and excluded from the analysis for animal welfare reasons (Table 2). Consequently, the final sample sizes were 17 rats in the CS group, 17 rats in the NP group, and 16 rats in the IB group.

**TABLE 1:** Implanted materials.

Group	Grafting method	N
IB	IB (80 mg)	18
CS	CS containing rhBMP-2 (10 μg, 0.10 mg/mL)	18
NP	NP containing rhBMP-2 (10 μg, 0.10 mg/mL)	18

**TABLE 2:** Samples excluded for analysis.

Group	Reason for exclusion	Time for sacrifice after second operation
IB	Complete dislocation of screws	2 weeks
IB	Complete dislocation of screws	4 weeks
CS	Femoral fracture occurring during fixation surgery	0 days
NP	Death due to wound infection	2 weeks

#### 2.2.2 Anesthesia and housing conditions

The rats were anesthetized with a combination of 0.15 mg/kg medetomidine (Nippon Zenyaku Kogyo Co., Ltd., Fukushima, Japan), 2.0 mg/kg midazolam (Astellas Pharma, Inc., Tokyo, Japan), and 2.5 mg/kg butorphanol (Meiji Seika Pharma Co., Ltd., Tokyo, Japan), administered intramuscularly. This anesthesia protocol was used during surgery and *in vivo* CT imaging. For post-operative analgesia, 2.5 mg/kg butorphanol was administered subcutaneously every 12 h for 2 days. After the first and second surgeries (see details below), the rats were housed in separate cages to prevent them from attacking each other, with no weight-bearing restriction, and were provided with food and water *ad libitum*; their condition was monitored daily. Antibiotics (penicillin G potassium, 22,000 units/kg; Meiji Seika Pharma Co., Ltd.) were administered subcutaneously every 12 h for 2 days post-operatively. The rats were euthanized at 6 weeks post-operation using carbon dioxide inhalation in accordance with the AVMA Guidelines for the Euthanasia of Animals (2020 edition), and their femurs were extracted. The operated femoral segments were fixed in 10% formalin.

### 2.3 Surgical procedures (two-stage surgeries)

#### 2.3.1 Creation of nonunion (first operation)

**FIGURE 2:**
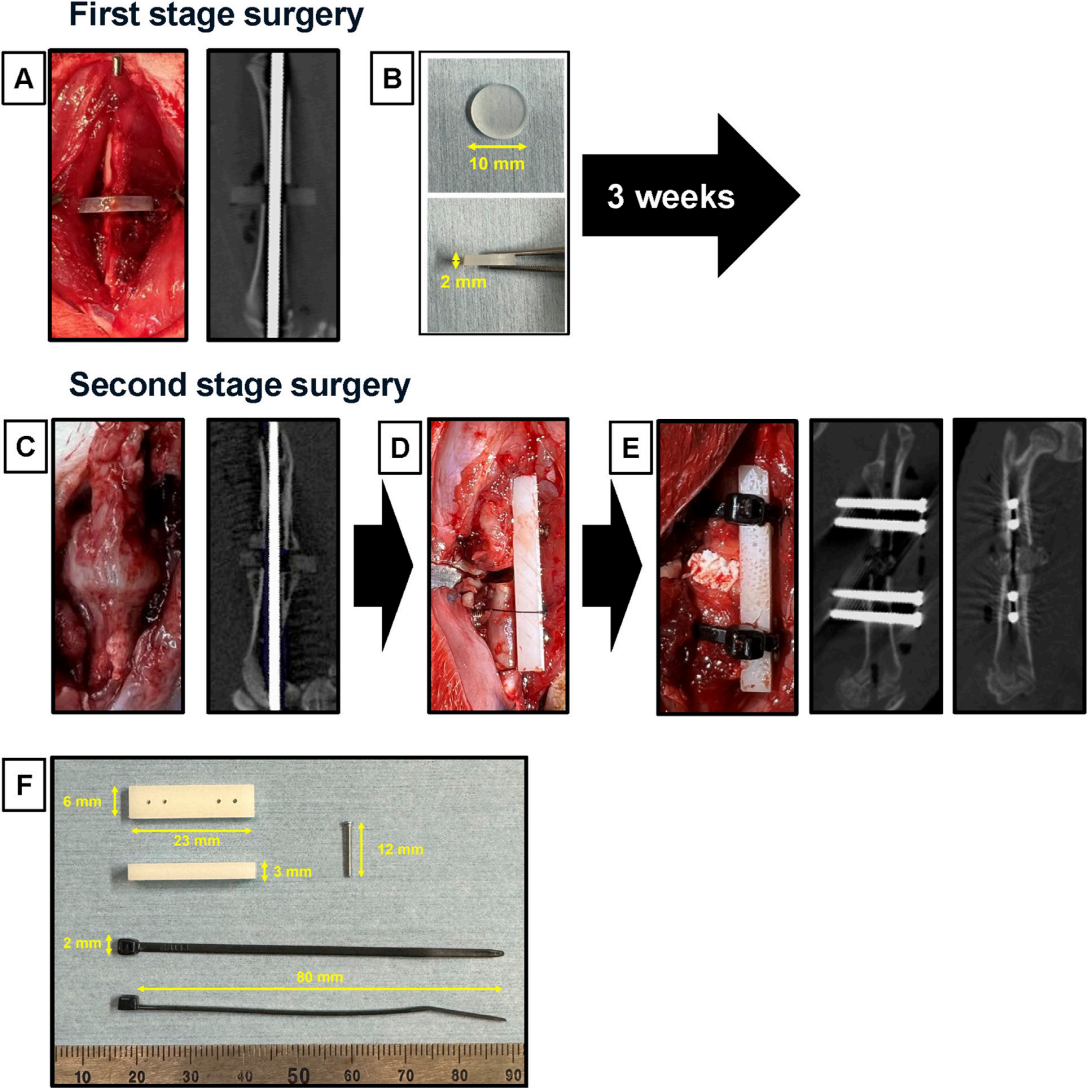
Surgical procedures of the modified nonunion model. **(A)** In the first operation, an intramedullary Kirschner wire was inserted, with a silicon disk interposed at the mid-diaphysis fracture site. An *in vivo* micro-CT image of the femur was taken immediately after the first operation. **(B)** The silicon disk was 10 mm in diameter and 2 mm thick. **(C)** During the second operation, macroscopic appearance and *in vivo* micro-CT images were captured 3 weeks after the first operation, showing the formation of a callus and scar tissue around the fracture site. **(D)** The procedure involved converting from intramedullary fixation to plate fixation while maintaining the 2 mm defect. **(E)** An iliac bone graft was placed into the gap. *In vivo* micro-CT images were also captured immediately after the second operation. **(F)** The materials used included a polyoxymethylene plate (23 mm length × 6 mm width × 3 mm height), a stainless-steel screw (φ1.2 × 12 mm), and nylon ligature bands (70 mm long × 2 mm wide).

This novel nonunion model was created by modifying the model created in a previous study (Tateiwa et al., 2024). We exposed the right femur of each animal using lateral and transverse approaches. A bone saw was used to create a mid-diaphyseal fracture. The knee joint was then exposed via the medial parapatellar approach, an intramedullary Kirschner wire (K-wire; 1.2 mm diameter) was inserted in a retrograde manner from the distal center of the femur (Figure 2A), and a silicone disk (10 mm diameter; 2 mm thick) (AS ONE, Osaka, Japan) (Figure 2B) was interposed between the fractured ends. To induce instability and promote nonunion, we used intramedullary fixation with a K-wire. After cutting the distal end of the K-wire, the muscle and skin were sutured.

#### 2.3.2 Intervention for nonunion (second operation)

The second-stage surgery was performed 3 weeks after the first surgery. The right femur was re-exposed via a lateral approach (Figure 2C). After removing fracture-site scar tissue, K-wire and silicone disk, the femur was performed internal fixation with a polyoxymethylene plate (23 mm long × 6 mm wide × 3 mm high; Matech Co., Ltd. Japan) and four stainless-steel screws (ø1.2 × 14 mm; Matsumoto Industry Co. Ltd., Chiba, Japan). The plate was positioned on the femur, and holes were drilled with a φ1 mm drill (MonotaRO Co., Ltd.). Then, we inserted four stainless-steel screws to fix the plate (Figure 2D). In cases where the stability of the plate fixation was considered insufficient, reinforcement was performed using nylon ligature bands (70 mm long × 2 mm wide; Star Tech.com Co. Ltd., Tokyo, Japan) (Figure 2F). The fracture ends were freshened using a high-speed bar to remove soft scar tissue. Then, each material was implanted into the gap (Figure 2E).

### 2.4 Preparation of grafting materials

#### 2.4.1 Allogenic iliac cancellous bone

The allogenic iliac cancellous bone was harvested from four donor SD rats, 8 weeks old, immediately before surgery. The volume of IB grafting for each defect space was set to 100 mm^3^ (80 mg).

#### 2.4.2 Collagen sponge with rhBMP-2

A disk-shaped collagen sponge (8.0 mm in diameter and 3 mm thick) was prepared by punching out an absorbable collagen sponge (CS; Colla Tape, Zimmer Dental, Carlsbad, CA, United States; Figure 1C). A total of 100 μL of phosphate-buffered saline containing 10 μg of rhBMP-2 (Daewoong Pharmaceutical Co., Ltd., Seoul, Korea; Lee, 2011) was added to each CS (0.10 mg/mL), which was then freeze dried in a benchtop freeze dryer (Free Zone 2.5, Labconco, Kansas City, MO, United States).

#### 2.4.3 HA/β-TCP/hydrogel composite with rhBMP-2

Materials for the NP group, including HA granules, β-TCP microsphere/poloxamer 407 hydrogel (Veyries et al., 1999; Dumortier et al., 2006), and rhBMP-2, were kindly provided by CGBio Co., Ltd. The hydrogel was mixed with β-TCP microspheres at a 1:1 weight ratio. *Escherichia coli*-derived rhBMP-2 (Daewoong Pharmaceutical Co., Ltd., Seoul, Korea) (Lee, 2011) was dissolved in saline to prepare a concentration of 1 μg/μL. Subsequently, HA granules were combined with the β-TCP/hydrogel mixture at a 3:2 weight ratio in a special mixing syringe to form homogeneous clay-like composites. These composites were molded to a volume of 100 mm³ (80 mg) (Figure 1A). Just before implantation, 10 μL of the prepared rhBMP-2 was added and mixed into the composite.

### 2.5 X-ray analysis

Radiographs were taken using a SOFTEX M-60W (SOFTEX Corp., Ebina-shi, Kanagawa, Japan). The radiographs were then evaluated by three blinded independent observers using a previously described scoring system: 0 (minimal to no evidence of new bone formation), 1 (evidence of bone formation, complete healing questionable), and 2 (solid-appearing bone, complete healing; Peterson et al., 2005). Each observer’s scores were summed, resulting in a total score ranging from 0 to 6.

### 2.6 *In vivo* micro-CT analysis

*In vivo* micro-CT was performed immediately after surgery and every week after surgery until euthanasia (6 weeks). The treated rat femurs were scanned using micro-CT (R_mCT; Rigaku Mechatronics, Tokyo, Japan) at a resolution of 59 μm/voxel *in vivo*, and the data were collected at 90 kV and 160 μA. Visualization and data reconstruction were conducted using a SimpleViewer (Rigaku Corporation, Tokyo, Japan).

### 2.7 *Ex vivo* micro-CT analysis

After the rats were sacrificed, the extracted rat femurs were scanned using high-resolution micro-computed tomography (micro-CT) (SkyScan 1,272, Bruker Corporation, Billerica, MA, United States) at a source voltage and current of 80 kV and 125 μA, respectively. The detailed acquisition settings, image processing parameters, and segmentation methods are shown in the appendix (Supplementary Material 1). Bone union was determined by the bony continuity of the proximal and distal fracture ends. In successfully fused samples, bone volume (BV), tissue volume (TV), BV/TV ratio, and BMD were measured using CT-Analyzer software (Blue Scientific, Ltd., Cambridge, United Kingdom).

The region of interest (ROI) for BMD measurement was defined on CT images as follows. In the coronal plane, the center of the bone was determined based on the screw insertion points. In the sagittal plane, the center of the femur was determined. The defect area was identified as a 2 mm range, anterior and posterior to the defect. The ROI for BMD measurement was set within a 1 mm vertical and horizontal range from the center of the bone (Supplementary Figure 2).

### 2.8 Histological analysis

Tissue samples were fixed in formalin, decalcified in 10% ethylenediaminetetraacetic acid (EDTA), dehydrated using ethanol, embedded in paraffin wax, and serially sectioned at 3-μm thickness. Hematoxylin and eosin staining and Safranin-O/fast green staining was performed according to standard protocols. A 3 mm × 2 mm ROI was set in the newly formed fusion mass. The new bone area (red) was color coded using the ImageJ software (version 1.52q, US National Institutes of Health, Bethesda, Maryland, United States), and the percentage of the newly formed bone area in the ROI was calculated.

### 2.9 Statistical analysis

ANOVA and Tukey’s HSD Post-Hoc test was used to compare bone fusion rates and bone healing scores. Unpaired Student’s *t*-test was used to compare bone morphometric analysis results. Data were analyzed using GraphPad Prism 8.0 and were expressed as the mean ± standard deviation. Statistical significance was set at *p* < 0.05.

## 3 Results

### 3.1 Bone fusion analysis by micro-CT and X-ray

**FIGURE 3:**
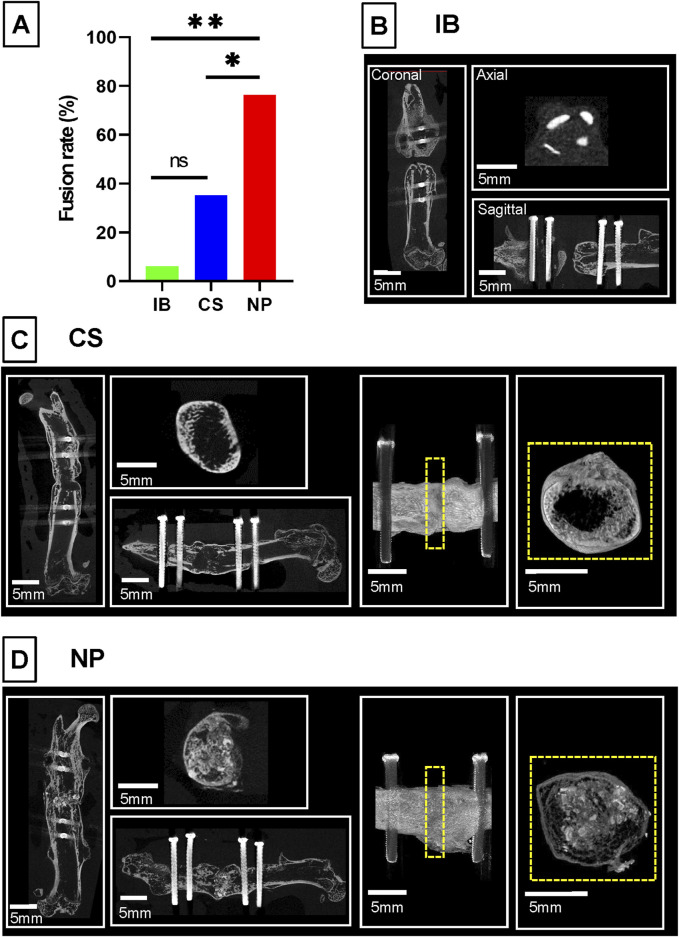
Bone fusion analysis by micro-CT. Representative micro-computed tomography images and union rates of different intervention groups. **(A)** In the IB group, bone union was not achieved, and almost all the grafted bone was resorbed. **(B)** In the CS group, bone union was achieved with scant new bone. **(C)** In the NP group, bone union was achieved with dense new bone tissue. **(D)** The union rate of the NP group (n = 13/17, 76.5%) was significantly higher than that in the CS group (n = 6/17, 35.3%) and IB group (n = 1/16, 6.3%; *p < 0.05 by ANOVA and Tukey’s HSD Post-Hoc test). BMP, bone morphogenetic protein; IB, iliac bone.

At 6 weeks, the fusion rates evaluated by *ex vivo* micro-CT were 6.3% in the IB group, 35.3% in the CS group, and 76.5% in the NP group. The fusion rate in the NP group was significantly higher than that in both the IB group (*p* = 0.001) and CS group (*p* < 0.001) (Figure 3A). In the IB group, almost all the grafted bone was resorbed (Figure 3B). In the CS group, 35% of the treated femurs showed bony continuity, but the fusion mass was composed of a thin cortical shell, and the amount of new bone inside the fusion mass was small (Figure 3C). In the NP group, bone union was achieved with dense new bone tissue (Figure 3D).

The X-ray bone healing scores were 0.88 ± 1.41 in the IB group, 2.35 ± 2.74 in the CS group, and 4.24 ± 1.99 in the NP group. The NP group score was significantly higher than that of the CS and IB groups (*p* < 0.05; Supplementary Figure 3).

### 3.2 Changes over time in the treated nonunion sites using *in vivo* micro-CT

**FIGURE 4:**
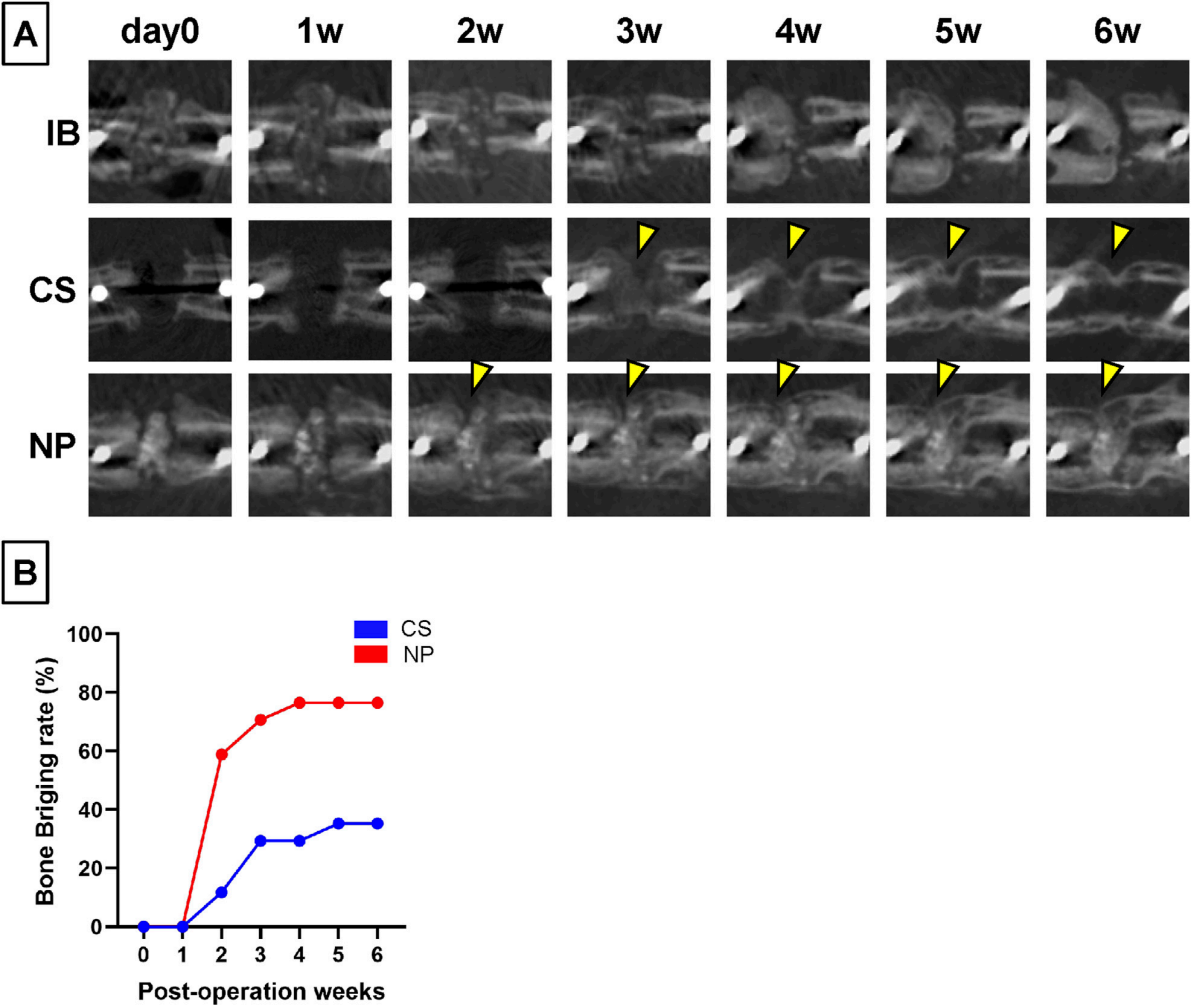
Changes over time in the treated nonunion sites using micro-CT. **(A)** In the IB group, the grafted bone was absorbed over time and almost disappeared at 6 weeks post-operation. In the CS group, new bone formation inside the fracture gap was observed 2 weeks post-operation. New bone formation toward the nonunion was observed (yellow arrowheads). However, the bone formation at the gap was small and scant even 6 weeks post-operation. In the NP groups, the density of HA and β-TCP decreased over time. **(B)** New bone formation was observed at 2 weeks post-operation, and the density of the new bone increased over time until 6 weeks post-operation.

In the IB group, the dense portion of the grafted bone was resorbed over time, the marrow cavity was occluded, and osteosclerosis was observed at the fracture ends (Figure 4A; IB group). In the CS groups, new bone formation was observed from 3 weeks after the implantation and bony continuity from 5 weeks, but the bridging new bone was thin, and BV was low (Figure 4A; CS group, yellow arrowheads). In contrast, in the NP groups, new bone formation was observed around the shadow reflecting HA or b-TCP from 2 weeks postoperatively; the new bone intensity and volume increased over time, and the nonunion area was filled with dense new bone (Figure 4A; NP group). The bone bridging rates were investigated for the NP and CS groups using *in vivo* CT. In the NP group, the bone bridging rate began to increase significantly from week 2, reaching 58.8%, and continued to rise, reaching a plateau at 76.5% by week 4 (and remained constant through week 6). In contrast, the CS group showed a delayed increase in bone bridging, starting to rise from week 3 with a rate of 29.4% and gradually increasing to 35.3% by week 5 and maintaining this rate through week 6. After 4 weeks post-operation, a significant difference in bone bridging rates between the NP and CS groups was observed (Fisher’s exact test: p = 0.016 at 4 weeks, p = 0.038 at 5 weeks; Figure 4B).

### 3.3 Microstructural analysis of fused nonunion gaps by micro-CT

**FIGURE 5:**
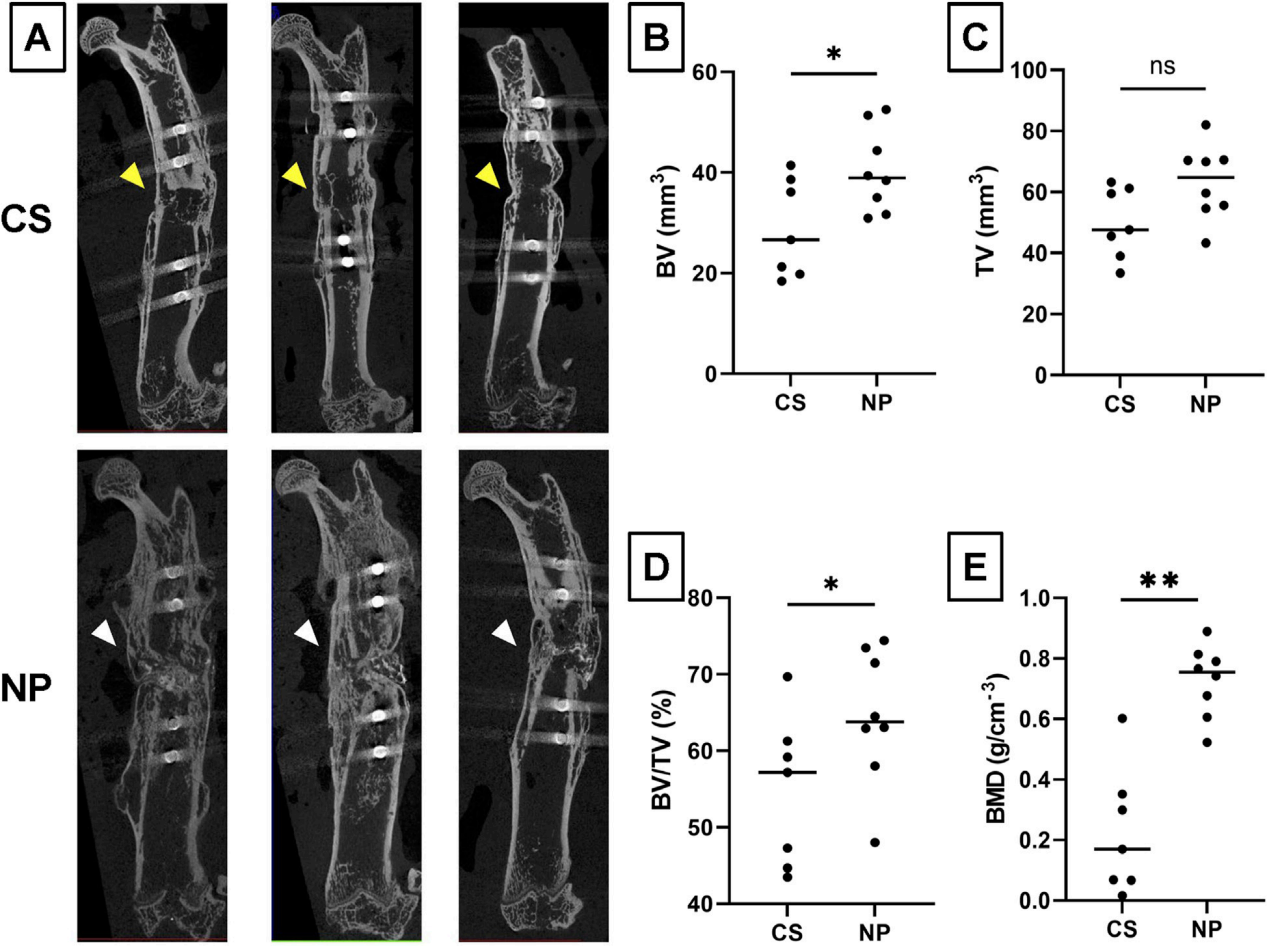
Microstructural analysis of fused nonunion gaps by micro-CT. **(A)** Representative micro-CT images of the NP and CS groups. **(B)** Bone Volume (BV) measurements for the NP and CS groups. **(C)** Tissue Volume (TV) measurements for the NP and CS groups. **(D)** The Bone Volume/Total Volume (BV/TV) ratio for the NP and CS groups. **(E)** Bone Mineral Density (BMD) for the NP and CS groups. NP group, n = 9; CS group, n = 7; **p < 0.01, *p < 0.05; ns, not significant by Student’s *t*-test.

Microstructural analysis of fused segments using *ex vivo* micro-CT was performed using samples in the CS and NP groups in which bone fusion was achieved (Figure 5A). The BV/TV and BMD of the new bone in the NP group were significantly higher than those in the CS group (BV, *p* = 0.027; TV, *p* = 0.051; BV/TV, *p* = 0.031; and BMD, *p* < 0.0001; Student’s *t*-test), suggesting that NP allows better quality bone formation than CS (Figures 5B–E).

### 3.4 Histological analysis

**FIGURE 6:**
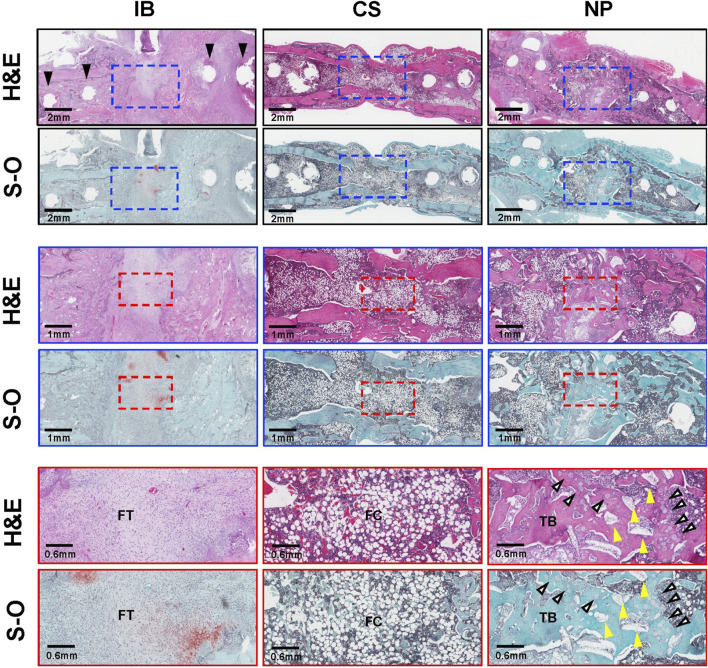
Histological analysis. Histological images of treated segments at 6 weeks post-operation. Hematoxylin and eosin (H&E) staining of treated segments. The IB group filled the fracture gap with fibrous tissue (FT). Black arrows indicate screw holes. In the CS group, the fracture gap was filled with structurally abnormal bone with scant trabecular bone (TB) and abundant fatty cells (FCs). In the NP group, the fracture gap was filled with new bone with a TB structure, a small amount of HA granules remained in the fracture gap (yellow arrowheads), and some of the β-TCP microspheres were resorbed (white arrows) and replaced by new bone. BMP, bone morphogenetic protein; FC, fatty cells; FT, fibrous tissue; H&E, hematoxylin and eosin; IB, iliac bone; S-O, Safranin-O; TB, trabecular bone.

Histological analysis showed that, in the IB group, the fracture gaps were filled with fibrous and fibrocartilage tissue. In the CS group, although bridging thin cortical bone formation was present, the interior of the new bone was filled with fatty marrow, and little trabecular bone structure was observed. In the NP group, the fracture gap was filled with new bone with abundant trabecular bone structure, and a small amount of HA granules and β-TCP microspheres remained and were incorporated into the newly formed bone (Figure 6).

### 3.5 Histological quantification of new bone area

**FIGURE 7:**
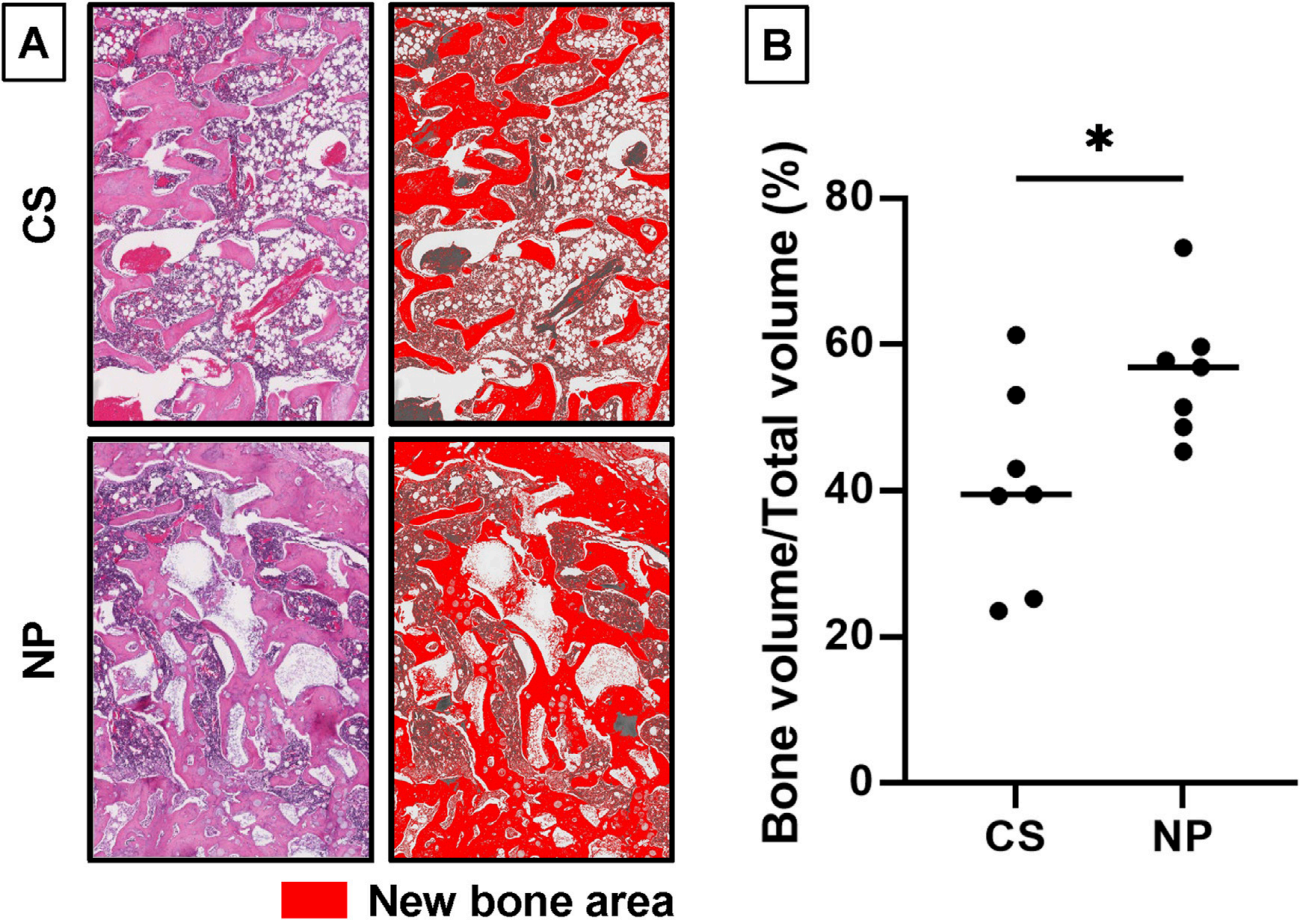
Quantification of new bone area using histological sections. **(A)** The new bone areas within the newly formed fusion mass in histological sections were compared. A 1.5 × 2 mm^2^ region of interest (ROI) in the fracture gap was extracted. The new bone area was color-coded using ImageJ software. (version 1.52q, US National Institutes of Health; https://imagej.nih.gov/ij/). **(B)** The percentage of the newly formed bone area in the ROI was significantly higher in the NP groups than in the CS groups (NP group, n = 9; CS group, n = 7; *p = 0.0284) (CS group, 40.7%; NP group, 56.2%; data represent mean ± SD; *p < 0.05 by Student’s *t*-test).

In addition to micro-CT, new bone formation was quantitatively examined using histological sections Figure 7A). The percentage of the new bone area was significantly higher in the NP groups (56.2%) than in the CS group (40.7%; *p* = 0.0284, *p < 0.05; Figure 7B).

## 4 Discussion

In this study using an animal model of femoral nonunion, the NP (HA/β-TCP/hydrogel composite with rhBMP-2) demonstrated significant improvements in the bone fusion rate and bone microstructure, as evaluated by micro-CT. Histological analysis further confirmed that using NP led to denser bone formation compared with conventional CS. These findings suggested that the HA/β-TCP/hydrogel composite was a promising new option for the treatment of nonunion in which the surrounding tissue environment was very harsh for bone tissue regeneration.

Several factors may have contributed to the superior effect of the HA/β-TCP/hydrogel composite over CS in nonunion treatment.

First, the BMP-2 sustained release effect of the HA/β-TCP/hydrogel composite was crucial. CS releases most of its BMPs within 1-day post-implantation (burst release), which was insufficient for inducing an osteogenic response in the surrounding tissue (Yang et al., 2012; Bhakta et al., 2013; Krishnan et al., 2017). In contrast, the HA/β-TCP/hydrogel composite released BMPs over 3 weeks, as shown in previous studies (Lee et al., 2014; Lyu and Lee, 2020; Tateiwa et al., 2021). This sustained release allowed continuous bone formation (Poldervaart et al., 2013; Seo et al., 2015), even in poor biological environments such as nonunion, in which bone formation takes longer (Brinker and O’Connor, 2016; Rupp et al., 2018). In this study, the HA/β-TCP/hydrogel composite group exhibited bone formation around the implant as early as 2 weeks postoperatively, with sustained bone formation and increased bone fusion rates observed up to 6 weeks. Conversely, the CS group showed initial bone formation at 2 weeks postoperatively, but the rate of increase in bone formation and bone healing diminished thereafter, resulting in a lower amount of new bone formation.

Second, the HA and β-TCP components in the HA/β-TCP/hydrogel composite provided a scaffold for bone formation. The composite combined two scaffolds with different *in vivo* degradation rates: HA and β-TCP (Tateiwa et al., 2021). At 6 weeks postoperatively, tissue evaluation revealed robust new bone formation around the low-biodegradable HA, while the β-TCP was degraded and replaced by new bone.

Third, the HA/β-TCP/hydrogel composite offered excellent handling characteristics due to its putty properties and increased mechanical strength from including HA. Previous reports have indicated that CS has poor handling properties and cannot be placed adequately at the target site (Lee et al., 2001; Schmidmaier et al., 2007). Due to HA, the mechanical strength of the HA/β-TCP/hydrogel composite prevented deformation by compression from surrounding tissues. This property aids in maintaining a scaffold for spatial control of bone formation and facilitated the migration of osteogenic cells to the graft by conforming to the bone shape.

Despite these promising results, this study has limitations. First, the femoral nonunion model did not evaluate BMP-related side effects, as soft tissue around the femur was thick. However, previous reports have demonstrated that the HA/β-TCP/hydrogel composite reduced inflammation-related adverse events compared with CS in a rat caudal vertebral fusion model, which suggested similar reducing effects on adverse events in the femur (Tateiwa et al., 2021; Nakagawa et al., 2022). Second, HA in NP takes longer to be absorbed *in vivo* than collagen sponge. In fact, some HA granules remained in the newly formed fusion mass. In nonunion areas where conditions for bone formation are poor, we believe that the long-term remaining scaffold for bone formation is advantageous for the acquisition of bone fusion. Third, we did not include biomechanical assessments such as finite element (FE) analysis or mechanical testing of the regenerated bone. While FE analysis can provide valuable insights into the mechanical properties and strength of newly formed bone (Dailey et al., 2023; Inglis et al., 2022), accurately modeling the small size and complex geometry of the rat femur after two surgeries is technically challenging and beyond our current resources. Therefore, we were unable to conduct these assessments. Future studies should incorporate biomechanical evaluations, including FE analysis or mechanical testing, to better understand the functional restoration and mechanical integrity of the regenerated bone. This would provide important data on the strength and durability of the new bone, which are critical for translating these findings into clinical applications.

## 5 Conclusion

In a rat femoral nonunion model, the HA/β-TCP/hydrogel composite combined with BMP-2 enhanced the bone fusion rate and the quality of newly formed bone to the CS containing BMP-2. The HA/β-TCP/hydrogel composite is a promising alternative carrier material for BMP-2 compared with CS carriers for the treatment of nonunion.

## Data Availability

The raw data supporting the conclusions of this article will be made available by the authors, without undue reservation.
