# Supplementary figures and images for: rhBMP-2-loaded hydroxyapatite/beta-tricalcium phosphate microsphere/hydrogel composite promotes bone regeneration in a novel rat femoral nonunion model
Source: Front Bioeng Biotechnol. 2024 Oct 7;12:1461260. doi: 10.3389/fbioe.2024.1461260 (PMC11492530; doi:10.3389/fbioe.2024.1461260)

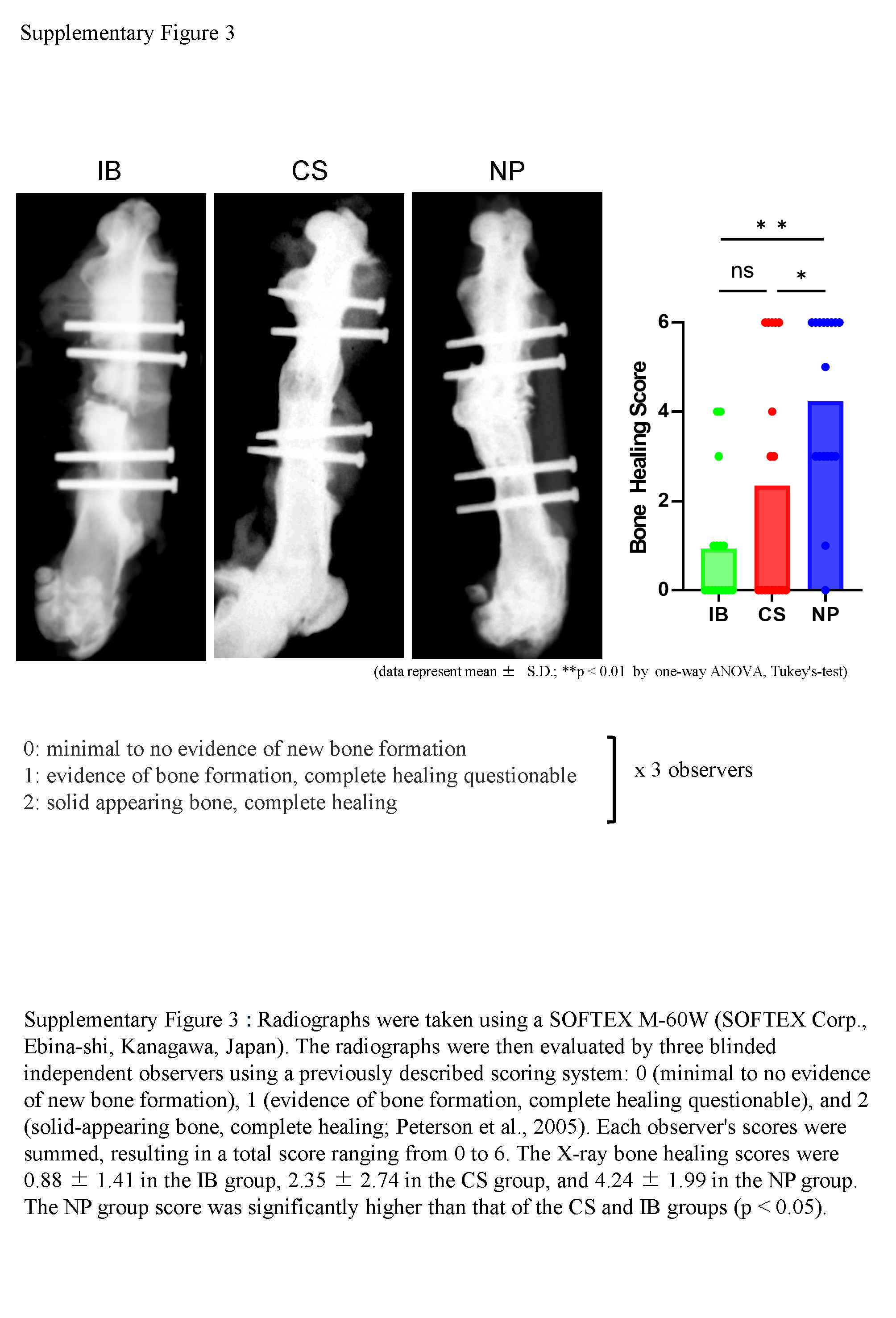

Supplement: Supplementary file 1 [file Image3.TIF]

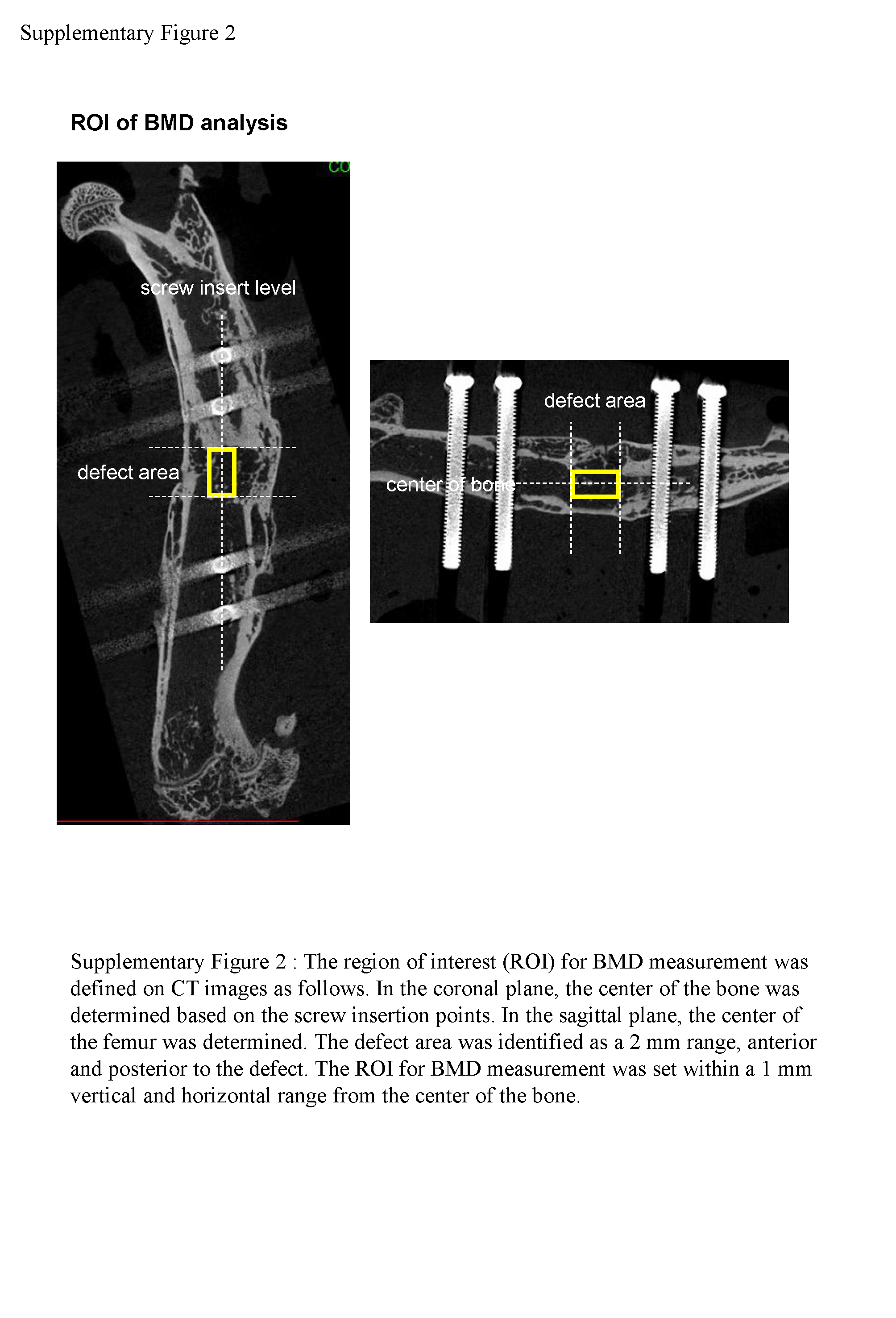

Supplement: Supplementary file 2 [file Image2.TIF]

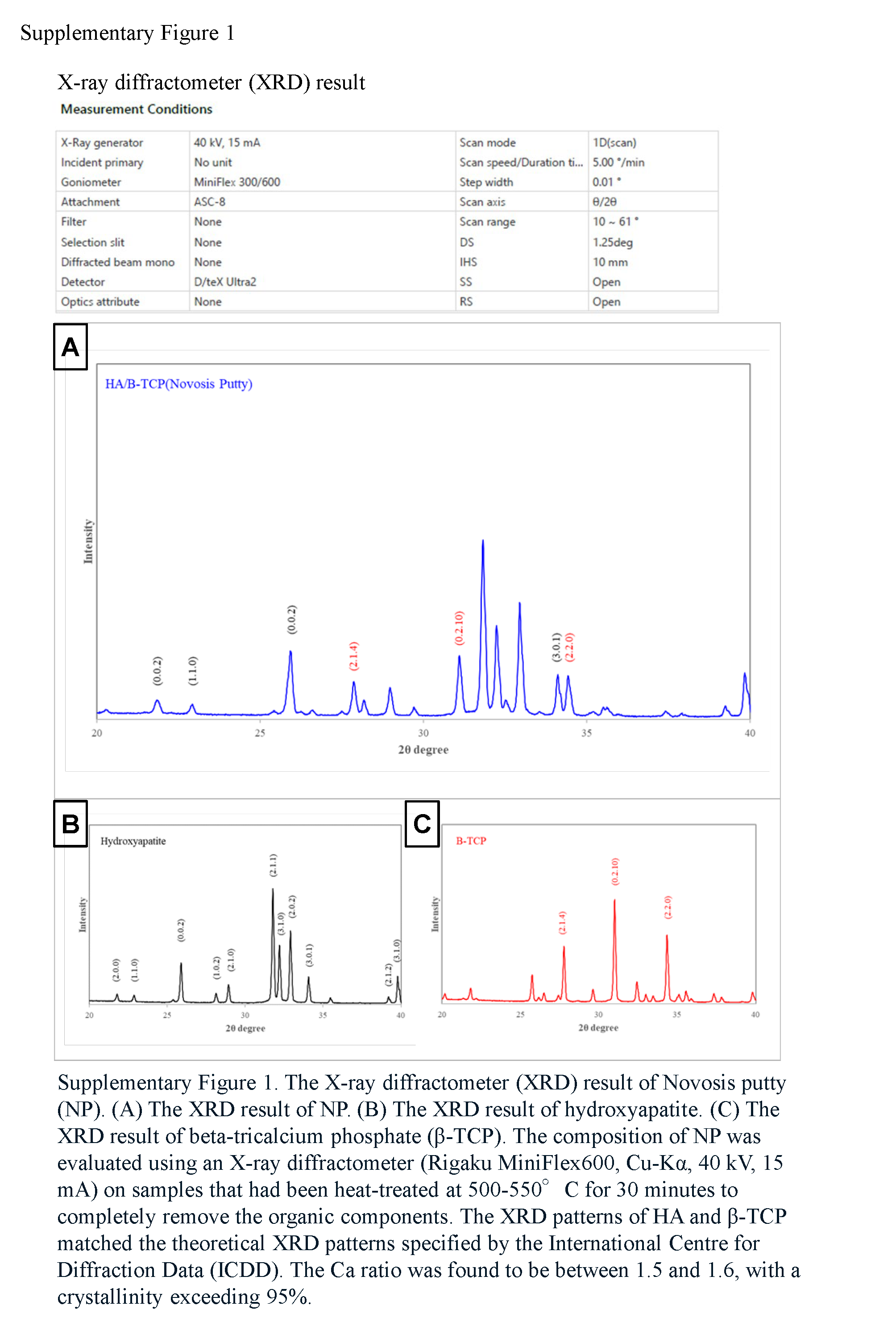

Supplement: Supplementary file 3 [file Image1.TIF]
