# Supplementary material for: rhBMP-2-loaded hydroxyapatite/beta-tricalcium phosphate microsphere/hydrogel composite promotes bone regeneration in a novel rat femoral nonunion model
Source: Front Bioeng Biotechnol. 2024 Oct 7;12:1461260. doi: 10.3389/fbioe.2024.1461260 (PMC11492530; doi:10.3389/fbioe.2024.1461260)
Supplement: Supplementary file 4 [file DataSheet1.PDF]

Supplementary information 1

Micro-CT Settings

Acquisition Settings

Number of Projections: 190
Integration Time (Exposure Time): 414 milliseconds
Resolution: The camera pixel size was 9.0 μm, with a scaled image pixel size of 26.441509 μm.
Image Format: TIFF, 16-bit depth
Rotation Step: 1.000 degree
Source Voltage: 70 kV
Source Current: 142 μA
Filter: Al 0.5 mm

Image Processing Parameters

Reconstruction Program: NRecon, Version 1.7.4.2
Filter Type: Hamming (Alpha=0.54)
Smoothing: Gaussian kernel, 3 with smoothing kernel 2
Ring Artifact Correction: Level 6
Beam Hardening Correction: 25%
Reconstruction Angular Range: 190 degrees

Segmentation and Analysis

Manual contouring was performed for accurate measurements. Both β-TCP and HA were present in the new bone, distinguishing HA from the surrounding bone by an HU value exceeding 1500.
